# Risk factors of in-hospital mortality in patients with pneumocystis pneumonia diagnosed by metagenomics next-generation sequencing

**DOI:** 10.3389/fcimb.2022.994175

**Published:** 2022-09-26

**Authors:** Jun-Na Hou, Heng-Dao Liu, Qiu-Yue Tan, Feng-An Cao, Shi-Lei Wang, Meng-Ying Yao, Yang-Chao Zhao

**Affiliations:** ^1^ Department of Pulmonary, The First Affiliated Hospital of Zhengzhou University, Zhengzhou, China; ^2^ Department of Cardiology, Cardiovascular Center, Henan Key Laboratory of Hereditary Cardiovascular Diseases, The First Affiliated Hospital of Zhengzhou University, Zhengzhou, China; ^3^ Department of Extracorporeal Life Support Center, Department of Cardiac Surgery, The First Affiliated Hospital of Zhengzhou University, Zhengzhou, China

**Keywords:** metagenomic next-generation sequencing, pneumocystis pneumonia, in-hospital mortality, risk factor, lactate dehydrogenase (LDH)

## Abstract

**Objectives:**

The metagenomic next-generation sequencing (mNGS) test is useful for rapid and accurate detection and identification of pathogenic microorganisms. The aim of the present study was to investigate the factors associated with in-hospital mortality in pneumocystis pneumonia (PCP) patients with mNGS-assisted diagnosis.

**Methods:**

Our study enrolled 154 patients with mNGS-positive PCP from August 2018 to February 2022 at the First Affiliated Hospital of Zhengzhou University respectively. Patients were divided into the survivor group (n=98) and the death group (n=56) according to whether in-hospital death occurred. Baseline characteristics, patients’ pre-hospital symptoms and patients’ CT imaging performance during hospitalization were carefully compared between the two groups. Risk factors for the occurrence of in-hospital death were sought by selecting indicators that were significantly different between the two groups for modelling and performing multiple logistic regression analysis.

**Results:**

Compared with the in-hospital death patients, the survivors were younger and had higher levels of albumin (ALB) (age: 50.29 ± 14.63 years vs 59.39 ± 12.27 years, p<0.001; ALB: 32.24 ± 5.62 g/L vs 29.34 ± 5.42g/L, p=0.002; respectively), while the levels of lactate dehydrogenase (LDH) and C-reactive protein CRP were lower (LDH: 574.67 ± 421.24 U/L vs 960.80 ± 714.94 U/L, p=0.001; CRP: 54.97 ± 55.92 mg/L vs80.45 ± 73.26 mg/L, *p=*0.018; respectively). Multiple logistic regression analysis revealed that age, the baseline LDH and CRP levels were all positively associated with high in-hospital mortality [age: OR(95%CI): 1.115 (1.062-1.172), p<0.001; LDH: OR(95%CI): 1.002 (1.001-1.003), p<0.001; CRP: OR(95%CI): 1.008 (1.000-1.017), p=0.045; respectively] while the platelet counts was negatively associated with it [OR(95%CI): 0.986 (0.979-0.992), p<0.001].

**Conclusions:**

Old age, high baseline levels of LDH and CRP and low platelet counts were risk factors of the in-hospital mortality in mNGS positive PCP patients.

## Introduction

Pneumocystis carinii pneumonia (PCP) is a life-threatening opportunistic lung infection caused by the human-specific opportunistic fungus Pneumocystis carinii (Sokulska et al., 2015), commonly seen in immunocompromised individuals with a combination of human immunodeficiency virus (HIV) infection (CD4^+^ cell count <200 cells/mm^3^), tumors, organ transplants, autoimmune diseases and other causes (Yale and Limper, 1996; Thomas and Limper, 2004). The main symptoms of patients with PCP are fever, cough and dyspnea, and in severe cases respiratory failure may occur (Sepkowitz, 2002; Enomoto et al., 2010). Cell-mediated immunodeficiency and the use of glucocorticoids are among the strongest risk factors for the development of PCP (Sepkowitz et al., 1992; Yale and Limper, 1996). In recent years, the morbidity and mortality of non-HIV associated PCP has increased significantly with the increasing number of patients with oncology, organ transplantation and autoimmune diseases, as well as improvements in bronchoscopy and microbiological testing techniques (Sepkowitz, 2002). In addition, study has reported a significantly higher mortality rate in patients with HIV negative PCP than that in patients with HIV positive PCP (27% vs 4%) (Roux et al., 2014). Parameters such as old age, low CD4^+^ cell count, and high levels of lactate dehydrogenase (LDH) have been reported to be associated with poor prognosis in HIV-positive PCP patients (Antinori et al., 1993; Fernandez et al., 1995; Dworkin et al., 2001). It has also been suggested that low hemoglobin (HGB) levels, the presence of medical comorbidities and Kaposi’s sarcoma of the lung are predictors of death in patients with HIV-associated PCP (Walzer et al., 2008; Fei et al., 2009). In contrast, initial LDH levels, mixed infection and elevation of neutrophil/lymphocyte rate (NLR) were the useful predictors of in-hospital mortality in non-HIV-infected PCP patients (Schmidt et al., 2018; Duan et al., 2022).

The morbidity and mortality rate of PCP were still high, but the clinical manifestations of PCP patients were atypical, the culture of Pneumocystis was difficult that makes the microbiologically diagnose in immunocompromised patients difficult (Limper et al., 1989; Fishman, 2020). Metagenomics next-generation sequencing (mNGS) is a widely used molecular technology for clinical nucleic acid sequencing that allows unbiased detection of pathogens in a single test and is considered a promising technique for microbial identification in infectious diseases (Goldberg et al., 2015; Chiu and Miller, 2019; Han et al., 2020). Recently, several studies have demonstrated its advantages in detecting a wide range of pathogens in different clinical specimens (Zhang et al., 2020; Wu et al., 2020; Yue et al., 2021). In recent years, studies have also been conducted in immunocompromised patients with non-HIV-infected PCP, such as renal transplantation and hematological malignancies, and it is believed that mNGS has definite value in the early diagnosis of non-HIV-infected PCP (Chen et al., 2020; Liu et al., 2020; Duan et al., 2022).

Therefore, our present study enrolled 154 patients with positive alveolar lavage fluid (BALF) samples for Pneumocystis jirovecii tested by mNGS, irrespective of co-infection with HIV, to analyze factors that may affect short-term prognosis.

## Methods

### Study population

Our present study enrolled 154 patients with mNGS-positive PCP from August 2018 to February 2022 at the First Affiliated Hospital of Zhengzhou University. Patients were divided into the survivor group (n=98) and the death group (n=56) according to whether in-hospital death occurred.

**Figure 1:**
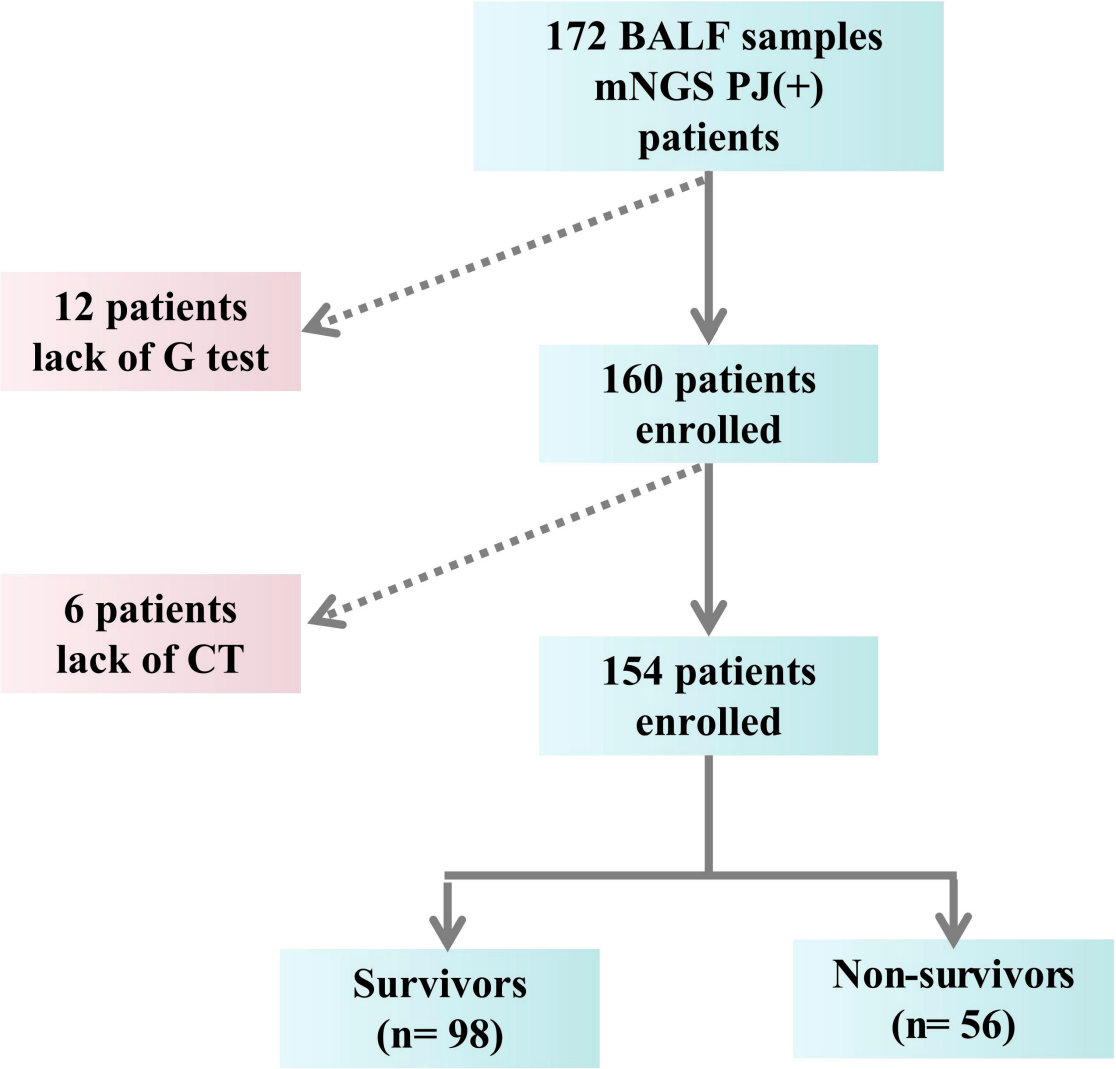
The flow chart.

Diagnosis of PCP must met the following criteria (Duan et al., 2022): (1) Clinical symptoms (cough, fever, or shortness of breath) and laboratory parameters [1,3-beta-D-glucan (BDG) test (G-test) and LDH] relevant to PCP; (2) Imaging findings compatible with PCP (ground glass opacity, present bilateral interstitial and alveolar infiltrates in perihilar areas); (3) Identifying the genetic sequences of Pneumocystis jirovecii by mNGS of BAL specimens. The exclusion criteria were: (1) Age < 18 years old; (2) Incomplete medical record (**Figure 1**). The endpoint of the study was in-hospital death.

### Data collection

For patients enrolled, the following parameters were recorded carefully: age, sex, previous history, comorbidities [hypertension, type 2 diabetes, coronary artery disease (CAD), and chronic obstructive pulmonary diseases (COPD)], primary disease, use of invasive ventilator, stay time of intensive care unit (ICU), the reads of mNGS, and baseline laboratory examinations. In addition, the patients’ pre-hospital symptoms and patients’ CT imaging performance during hospitalization were carefully recorded. The clinical data of the patients were obtained through the electronic medical record system.

All patients had bronchoscopy performed, and about 5ml BALF was used to extracted DNA by TIANamp Micro DNA Kit following the manufacturer’s instructions. DNA libraries were constructed by DNA fragmentation, end-repair, adapter ligation, and PCR amplification, followed by sequencing. Agilent 2100 bioanalyzer (Agilent, USA) and ABI StepOnePlus Realtime PCR System were used for quality control of DNA library, the qualified libraries were sequenced on NextSeq 550Dx platform (illumina,USA) using 75bp sequencing read length. Positive control and negative control were included in all assays. The lower limit of detection of the assay was estimated to be <50 copies/ml (as per the instruction of the manufacturer). DNA integrity of the samples was confirmed by the presence of internal control DNA.

### Statistical analysis

All collected data were statistically analyzed using SPSS 21.0 (Armonk, NY: IBM Corp.). Variables of two groups were compared using the Wilcoxon rank-sum test for continuous variables and the chi-square test for binary and categorical variables as appropriate. Multiple Logistic regression analyses were performed. P<0.05 indicates that the difference is statistically significant.

## Results

### Baseline characteristics of PCP patients

As shown in **Table 1**, PCP patients tend to have autoimmune diseases and hematological malignancies as their primary cause and are often treated with glucocorticoids and immunosuppressive drugs prior to admission to hospital. Compared with the in-hospital death patients, the survivors were younger and had higher levels of ALB and platelet, while the levels of LDH and CPR were lower [age: 50.29 ± 14.63 years vs 59.39 ± 12.27 years, p<0.001; ALB: 32.24 ± 5.62 g/L vs29.34 ± 5.42g/L, p=0.002; platelet: 213.92± 96.00 vs 154.93 ± 78.55, p<0.001; LDH: 574.67 ± 421.24 U/L vs 960.80 ± 714.94 U/L, p=0.001; C-reactive protein (CRP): 54.97 ± 55.92 mg/L vs80.45± 73.26 mg/L, p=0.018; respectively, **Table 1**].

**Table 1:** Baseline characteristics of PCP patients.

Variables	Survivors (n=98)	Non-survivors (n=56)	P Value
**Male**, (n%)	57 (58.2)	26 (46.4)	0.181
Age***, (y)	50.29 ± 14.63	59.39 ± 12.27	<0.001
**Previous History**, (n%)			
Surgery	50 (51.0)	22 (39.3)	0.182
Use of corticosteroids	83 (84.7)	43 (76.8)	0.278
Immunosuppressant	54 (55.1)	27 (48.2)	0.503
Chemotherapy*	17 (17.3)	3 (5.4)	0.045
**Comorbidities**, (n%)			0.317
Hypertension	31 (31.6)	29 (51.8)	
CAD	9 (9.2)	10 (17.9)	
Type 2 diabetes	18 (18.4)	13 (23.2)	
COPD	2 (2.0)	1 (1.8)	
**Primary disease**, (n%)			0.158
Organ transplantation	10 (10.2)	3 (5.4)	
Hematological malignancies	17 (17.3)	2 (3.6)	
Other malignant neoplasms	4 (4.1)	2 (3.6)	
Rheumatic and immune system diseases	37 (37.8)	29 (51.8)	
Renal disease	19 (19.4)	12 (21.4)	
Others	11 (11.2)	8 (14.3)	
**Invasive ventilator**, (n%)	5 (5.1)	6 (10.7)	0.209
**ICU time**, (days)	8.92 ± 9.19	9.23 ± 7.32	0.816
**Co-infecton**	39 (39.8)	28 (50)	0.240
**Baseline laboratory examinations**			
**White blood cell**, (10^9^/L)	9.89 ± 13.08	8.63 ± 4.35	0.485
**Hemoglobin**, (g/L)	109.77 ± 25.65	110.68 ± 20.52	0.820
Platelet***, (10^9^/L)	213.92 ± 96.00	154.93 ± 78.55	<0.001
**Neutrophils,** (10^9^/L)	7.60 ± 9.77	8.78 ± 9.41	0.467
Percentage of neutrophils*	79.45 ± 16.94	85.11 ± 14.68	0.039
**Lymphocytes**, (10^9^/L)	2.14 ± 11.58	0.69 ± 0.57	0.354
**ALT**, (U/L)	36.32 ± 56.14	29.74 ± 20.45	0.400
**AST**, (U/L)	34.91 ± 31.23	41.85 ± 19.72	0.138
Albumin**, (g/L)	32.24 ± 5.62	29.34 ± 5.42	0.002
**Total bilirubin**, (umol/L)	11.87 ± 34.34	24.55 ± 41.31	0.056
LDH**, (U/L)	574.67 ± 421.24	960.80 ± 714.94	0.001
**Creatinine**, (umol/L)	97.18 ± 98.95	123.29 ± 102.78	0.125
**Procalcitonin**, (ng/ml)	0.58 ± 1.89	1.66 ± 5.04	0.130
CRP*, (mg/L)	54.97 ± 55.92	80.45 ± 73.26	0.018
**ESR**, (mm/H)	50.28 ± 33.71	51.06 ± 38.35	0.899
**NT-pro-BNP**, (pg/ml)	252 (83-716)	1057 (378-3415)	0.079
**Lactate**, (mmol/L)	1.59 (1.00-2.24)	2.10 (1.20-3.40)	0.332
**G-test**, (ng/L)	214.16 ± 233.52	223.51 ± 196.46	0.801
**GMS staining**, (n%)	7 (7.1)	5 (8.9)	0.760
**CD4+T lymphocytes**, (cells/mm^3^)	222.97 ± 189.67	160.51 ± 131.84	0.065

The data was shown as the mean ± SD, median (interquartile 25-75) or n (percentage). ^*^ indicate significant difference (^*^ indicate p<0.05, ^**^ indicate p<0.01, and ^***^ indicate p<0.001). PCP= Pneumocystis jirovecii pneumonia; CAD= coronary heart disease; COPD= chronic obstructive pulmonary diseases; ICU= intensive care unit; ALT= Alanine transaminase; AST= Aspartate transaminase; LDH= Lactate dehydrogenase; CRP= C-reactive protein; ESR= erythrocyte sedimentation rate; CD 4= Cluster of Differentiation 4.

Moreover, the percentage of neutrophils between two groups showed significant difference (79.45 ± 16.94 vs85.11 ± 14.68, p=0.039, **Table 1**), in spite of the counts of white blood cell, neutrophils, lymphocytes and the levels of hemoglobin (all p>0.05, **Table 1**).

**Figure 2:**
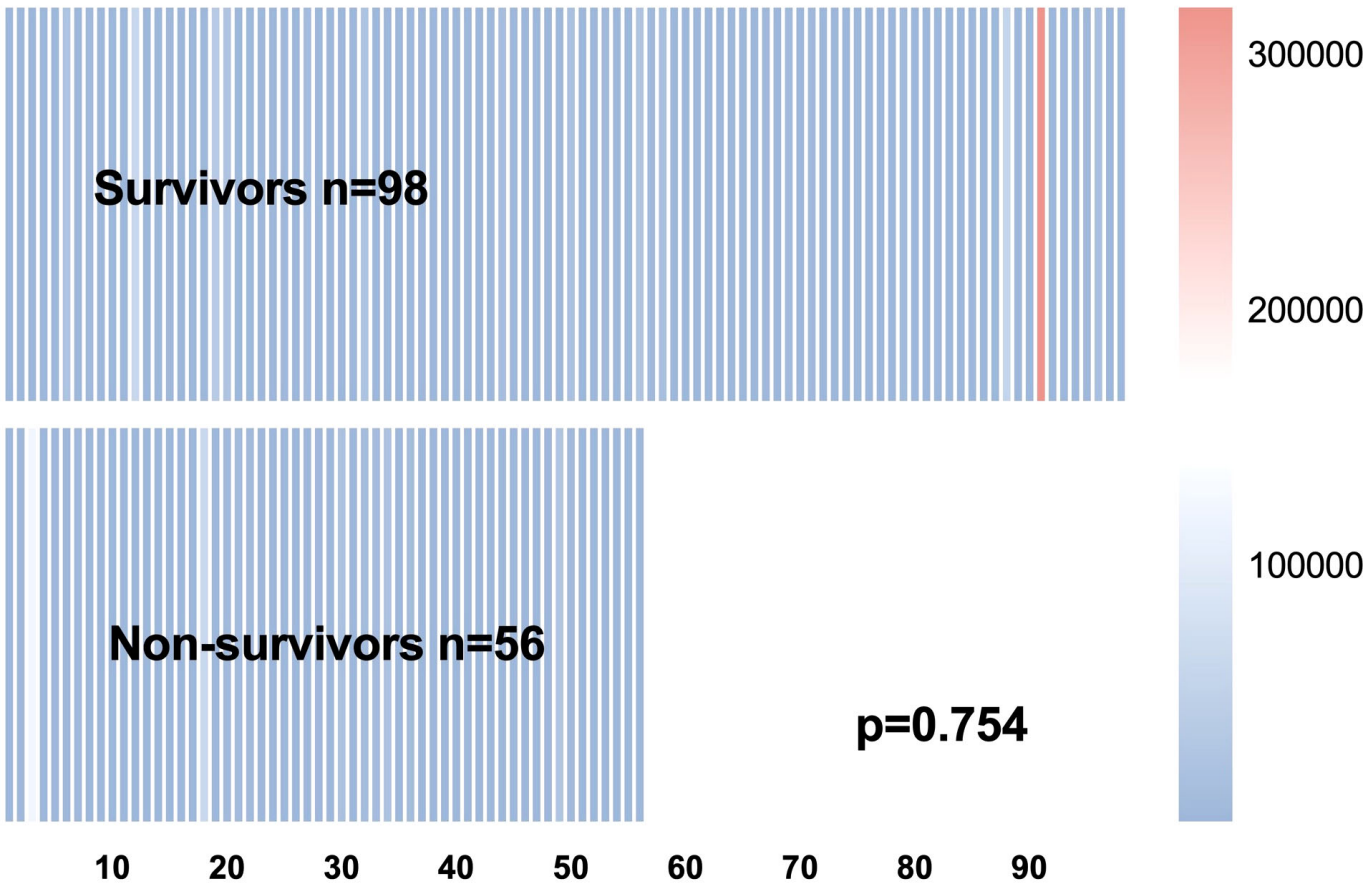
The heatmap of the reads number.

However, the mNGS reads of Pneumocystis jirovecii (p=0.754, **Figure 2**), the levels of PCT, G test and CD4^+^ T lymphocytes had shown no significantly differences between the survivors and non-survivors (all p>0.05, **Table 1**).

### Comparison of symptoms and CT films performances

Although there were no significant differences in the clinical symptoms and CT imaging features between the two groups of PCP patients (all p>0.05, **Tables 2**, **3**), we could still find that the most common clinical symptoms in PCP patients were fever, chest tightness, cough/sputum and fatigue, and the most common imaging features were ground glass images, pulmonary air sacs, hydrothorax, and consolidations.

**Table 2:** Comparison of symptoms between the two groups of patients.

Variables, (n%)	Survivors (n=98)	Non-survivors (n=56)	P Value
**Fever**	74 (75.5)	42 (75.0)	0.944
**Cough**	59 (60.2)	28 (50.0)	0.240
**Coughing sputum**	37 (37.8)	22 (39.3)	0.865
**Blood in sputum**	2 (2.0)	2 (3.6)	0.622
**Weakness**	47 (48.0)	23 (41.1)	0.501
**Sore throat**	4 (4.1)	3 (5.4)	0.705
**Muscle soreness**	10 (10.2)	3 (5.4)	0.377
**Dyspnea**	37 (37.8)	30 (53.6)	0.090
**Chest tightness**	61 (62.2)	39 (69.6)	0.385

The data was shown as n (percentage).

**Table 3:** Comparison of CT films of the two groups of patients.

Variables, (n%)	Survivors (n=98)	Non-survivors (n=56)	P Value
**Pulmonary air sacs**	18 (18.4)	10 (17.9)	0.925
**Lung consolidation**	23 (23.5)	18 (32.1)	0.259
**pulmonary cavity**	3 (3.1)	4 (7.1)	0.258
**Hydrothorax**	23 (23.5)	18 (32.1)	0.341
**Honeycomb sign**	5 (5.1)	3 (5.4)	0.958
**Pneumatothorax**	1 (1.0)	1 (1.8)	0.688
**Ground-glass opacity**	41 (41.8)	24 (42.9)	0.998

The data was shown as n (percentage). CT, computed tomography.

### Co-pathogens characteristics

The percentage of co-infections in PCP patients has shown no significant difference between the survivors and death patients (39.8% vs. 50%, p=0.240, **Table 1**). The possible pathogenic microorganisms detected in the BALF samples of the two groups of patients by mNGS technology were listed in the **Supplementary Table S1**. The most common co-pathogens in the survivors and death patients were cytomegalovirus (CMV) (20/20.4% vs. 11/19.6%, p=0.909) and Epstein-Barr virus (EBV) (13/13.3% vs. 13/23.2%, p=0.123). Haemophilus was the co-infected pathogen that differed between the two groups, and more was detected in the death group (3/3.1% vs. 10/17.9%, p=0.004, **Supplementary Table S1**). In addition, Moraxella (2/2.1%) and Nocardia (2/2.1%) detected in the survival group were not detected in the death group, while Pseudomonas aeruginosa (2/3.6%) and Staphylococcus aureus (1/1.8%) detected in the death group were not detected in the survival group. Other co-pathogens were Streptococcus, Aspergillus, Saccharomycete, Herpes simplex virus 1 (HSV1), Acinetobacter baumannii, Klebsiella Pneumoniae and Enterococcus (**Supplementary Table S1**).

### Predictors of in-hospital mortality

Based on comparing the indicators of the two groups (survivors and non-survivors), we selected age, use of chemotherapy, platelet counts, percentage of neutrophils, levels of albumin, levels of LDH and levels of CRP as variables. Multiple logistic regression analysis revealed that age, the baseline LDH and CRP levels were all positively associated with high in-hospital mortality [age: OR(95%CI): 1.115 (1.062-1.172), p<0.001; LDH: OR(95%CI): 1.002 (1.001-1.003), p<0.001; CRP: OR(95%CI): 1.008 (1.000-1.017), p=0.045; respectively, **Table 4**] while the platelet counts was negatively associated with it [OR(95%CI): 0.986 (0.979-0.992), p<0.001].

**Table 4:** Multivariate Logistic regression analysis of predictors of outcomes.

Variables	Multivariate analysis	
	OR (95%CI)	P Value
Age***	1.115 (1.062-1.172)	<0.001
**History of chemotherapy**	0.225 (0.039-1.296)	0.095
Platelet***	0.986 (0.979-0.992)	<0.001
**Percentage of neutrophils**	1.020 (0.987-1.054)	0.237
**Albumin**	0.980 (0.894-1.074)	0.669
LDH***	1.002 (1.001-1.003)	<0.001
CRP*	1.008 (1.000-1.017)	0.045

^*^ indicate significant difference (^*^ indicate p<0.05 and ^***^ indicate p<0.001).OR, odds ratio; CI, confidence; LDH, Lactate dehydrogenase; CRP, C-reactive protein.

## Discussion

In the present study, we analyzed PCP patients who tested positive for Pneumocystis jirovecii by mNGS and found that old age, low platelet, high LDH and CRP levels were independent predictors of in-hospital mortality in PCP patients.

Pneumocystis jirovecii cannot be routinely cultured and the positive rate for Pneumocystis jirovecii by GMS staining and direct microscopy is low, with a positive rate of 7.8% for GMS staining in our present study. mNGS has clear advantages in detecting opportunistic pathogens and mixed infections (Duan et al., 2022). Several recent studies using mNGS for diagnosis of Pneumocystis jirovecii have found that mNGS, an emerging microbiological test, has excellent sensitivity, even up to 100% (Pan et al., 2019; Wilson et al., 2019; Xie et al., 2019; Liu et al., 2020; Jiang et al., 2021). mNGS testing plays an important role in the diagnosis of PCP and may even affect the survival rate of patients with PCP (Duan et al., 2022). Therefore, we included patients with PCP who tested positive for Pneumocystis jirovecii by mNGS and further analyzed the possible factors influencing in-hospital mortality based on the early assistance of mNGS in the diagnosis of PCP.

One of the advantages of mNGS testing is the efficient detection of co-infections. It was found by other studies that several different types of pathogens could be found by mNGS such as human β-herpesvirus 5 and human γ-pesvirus 4 (Wang et al., 2022). In the present study, it also showed that the co-infections such as virus, fungus, gram-negative bacilli and gram-positive cocci were detected in the PCP patients. The proportion of Haemophilus in dead patients was higher, and Pseudomonas aeruginosa and Staphylococcus aureus were detected only in dead patients, which may be associated with poor prognosis. However, the percentage of co-infection has shown no significant difference between the two groups in our present study.

Patients with non-HIV-infected PCP are often immunocompromised, and in this study were predominantly patients with autoimmune diseases and hematological malignancies. Compared to HIV-infected PCP patients, non-HIV-infected patients have more complex primary illnesses, more comorbidities, require more ICU admissions, have shorter survival times and have higher mortality rates (Bitar et al., 2014; Kofteridis et al., 2014; Roux et al., 2014; Bienvenu et al., 2016). The initial levels of serum LDH at hospital admission have showed diagnostic evaluation in PCP. Boldt MJ et al. had claimed that in the differential diagnosis of PCP, LDH was better than the radiographic severity (Boldt and Bai, 1997). Sun J et al. had considered that LDH level, plasma IL-6/IL-10 ratio and IL-8 level predict the severity and the risk of death in PCP patients (Sun et al., 2016). A study that included a mixed population of HIV-positive and non-HIV-positive patients found that initial serum LDH levels at hospital admission not only contributed to diagnostic assessment but may be an independent predictor of survival. This has the potential to help clinicians identify early the most severe patients at high risk of an unfavorable outcome (Schmidt et al., 2018). Serum LDH has a strong relationship in the development of disease and the prognosis of PCP patients, which is very consistent with our findings. In addition, similar to LDH, this study found significantly higher levels of CRP in patients with PCP who died in-hospital than in survivors. Elevated levels of LDH and CRP in patients with PCP may be indicative of poor response to treatment and poor prognosis.

Previous studies demonstrated that younger PCP patients had a significant survival benefit (Boldt and Bai, 1997). Similarly, our findings found that survivors were younger and, in further analysis, age was found to be a risk factor for the short-term prognosis of PCP patients. In a recent study, the authors found that PCP patients in the death group had a persistent decrease in platelet count along with an increase in inflammatory markers (Sun et al., 2016). Our study did not observation dynamically at changes in platelet counts, but we found that baseline platelet levels were lower in PCP patients who died in-hospital, and that this difference was directly related to the prognosis of PCP patients. However, no statistically significant changes were observed in other inflammatory indicators included in our present study, such as PCT, erythrocyte sedimentation rate (ESR), CD4^+^ T lymphocyte, white blood cell, neutrophil, etc. Meanwhile, a study enrolled PCP patients with inflammatory bowel disease and found that hypoproteinemia could affects prognosis of the patients (Yoshida et al., 2019). Our study found a significant difference in albumin levels between the surviving and dying groups, but hypoalbuminemia was not a prognostic influence in this study. All these differences may be related to the small number of patients included.

Our study has several limitations. Firstly, the mNGS technique is highly sensitive in the diagnosis of pulmonary infections, although the diagnosis of PCP is a combination of clinical symptoms, test indicators and imaging manifestations, which still does not exclude the possibility of including patients with Pneumocystis jirovecii colonization. Secondly, this study counted in detail the medication used by patients prior to hospitalization, and the treatment regimen for patients after admission was adjusted in accordance with relevant guidelines and changes in their condition, and no statistics were kept on the medication used after admission. Thirdly, this study is a single-center, small sample size retrospective study and the conclusions drawn are of some reference value. Finally, This case-control study could not provide strong evidence for causal inference of in-hospital mortality in PCP patients, and further cohort study were needed.

## Conclusion

In conclusion, PCP is a fatal disease that occurred in immunocompromised patients with difference primary diseases. mNGS is a sensitive and useful diagnostic tool for identifying Pneumocystis jirovecii in PCP patients’ BALF samples. Advanced age, low platelet, high LDH and CRP levels were independent predictors of in-hospital mortality in PCP patients.

## Data availability statement

The data presented in the study are deposited in the EMBL database, accession number PRJEB55488.

## Ethics statement

The present study fully complied with the Declaration of Helsinki and was approved by the Ethics Committee of the First Affiliated Hospital of Zhengzhou University, Zhengzhou, China (no. 2022-KY-0273).

## Author contributions

J-NH and Y-CZ: Designed the study and wrote the first draft of the manuscript. H-DL, Q-YT, F-AC and S-LW: Verified data extraction, data analysis, and reviewed the manuscript. M-YY: Supervised the data acquisition, data analysis and interpretation. All authors read and approved the final manuscript.

## Funding

The work was supported by the National Natural Science Foundation of China Youth Fond (82000454) and Joint Project of Medical Science and Technology Research of Henan (LHGJ20190092).

## Conflict of interest

The authors declare that the research was conducted in the absence of any commercial or financial relationships that could be construed as a potential conflict of interest.

## Publisher’s note

All claims expressed in this article are solely those of the authors and do not necessarily represent those of their affiliated organizations, or those of the publisher, the editors and the reviewers. Any product that may be evaluated in this article, or claim that may be made by its manufacturer, is not guaranteed or endorsed by the publisher.
