# Supplementary material for: Risk factors of in-hospital mortality in patients with pneumocystis pneumonia diagnosed by metagenomics next-generation sequencing
Source: Front Cell Infect Microbiol. 2022 Sep 26;12:994175. doi: 10.3389/fcimb.2022.994175 (PMC9549864; doi:10.3389/fcimb.2022.994175)
Supplement: Supplementary file 1 [file Table_1.docx]

**Supplement Table 1. Comparison of therapy between the two groups of patients**

| **Variables**, (n%) | **Survivors**  (n=98) | **Non-survivors**  (n=56) | **P Value** |
| --- | --- | --- | --- |
| **Sulfamethoxazole** | 91 (92.9) | 51 (91.1) | 0.758 |
| **Antifungal therapy** | 77 (78.6) | 51 (91.1) | 0.072 |
| **Antibacterial therapy** | 98 (100) | 56 (100) | - |
| **Antiviral therapy** | 78 (79.6) | 48 (85.7) | 0.392 |

The data was shown as n (percentage).
